# Phosphatidic acid homeostasis regulated by a type-2 phosphatidic acid phosphatase represents a novel druggable target in malaria intervention

**DOI:** 10.1038/s41420-019-0187-1

**Published:** 2019-06-24

**Authors:** Raj Kumar Sah, Swati Garg, Poonam Dangi, Kalaiarasan Ponnusamy, Shailja Singh

**Affiliations:** 1 Special Center for Molecular Medicine, Jawaharlal Nehru University, New Delhi, India; 2 Department of Life Sciences, School of Natural Sciences, Shiv Nadar University, Gautam Buddh Nagar, Greater Noida, India; 3 School of Biotechnology, Jawaharlal Nehru University, New Delhi, India; 4 Present Address: Département de biochimie, Faculté de Médecine, Université de Montréal, Montreal, Canada

**Keywords:** Antiparasitic agents, Lipid signalling

## Abstract

Type-2 phosphatidic acid phosphatase (PAP2) a member of PAP2 superfamily mediates the conversion of phosphatidic acid (PA) to diacylglycerol (DAG) and thus plays a pivotal role in numerous cellular signaling processes in diverse organisms. An elevated level of intracellular PA is detrimental for the cell and induces cell death. In this study we identified and characterized a PAP2 homologue in *Plasmodium falciparum*, PfPAP2 and further elucidated its significance in regulation of PA homeostasis in parasite life cycle. PfPAP2 is expressed in the blood stage and harbors the canonical acid phosphatase domain (APD) with signature motifs. PfPAP2 catalyzes the dephosphorylation of PA to produce DAG and inorganic phosphate (P_i_). Propranolol, a generic inhibitor of PAP2, inhibited the phosphatase activity of PfPAP2 by binding to the active site of APD domain as evident by in silico docking and confirmed by surface plasmon resonance (SPR) analysis. Inhibition of native PfPAP2 by propranolol led to rise in intracellular PA mediating disruption of intracellular PA homeostasis in parasites. The propranolol mediated inhibition of PfPAP2 directed early secretion of a micronemal Perforin like Protein, PfPLP1 leading to untimely permeabilization and host cell egress. The merozoites following premature egress were non-invasive and were attenuated to invade erythrocytes and cannot continue next cycle growth. This study demonstrates that disruption of PA homeostasis can cause growth retardation in malaria parasites, and thus its master regulator, PfPAP2, can serve as a very good molecular target for antimalarial chemotherapeutic interventions.

## Introduction

Malaria continues to be a major global health problem despite of several exciting improvements in the treatment and prevention of malaria. This calls for the consistent efforts to develop an in-depth understanding of the events responsible for survival and pathogenesis of malaria parasite. The clinically significant stage of malaria parasite involves repeated cycles of erythrocyte invasion, multiplication and egress of next generation, invasive merozoites. Egress and invasion of merozoites is mediated by the parasite ligands residing within the apical secretory organelles such as micronemes and rhoptries^1,2^. Coordinated secretion of these parasite ligands from micronemes is essential for successful egress and invasion. This process is under high temporal and spatial regulation for the release of invasive merozoites and parasite survival. Ca^2+^ and cAMP have already been identified as important signaling second messengers that play a central role in regulation of microneme discharge from merozoites^3–5^. Therefore, the co-ordinated process of egress and invasion mediated by vesicular discharge is key for the cyclic progress of parasites in its intraerythrocytic life stage.

Recently, the role of lipids as second messengers was explored in a member of apicomplexan family, *Toxoplasma gondii*^6^. They showed that diacylglycerol (DAG) and its phosphorylated product Phosphatidic acid (PA) act as second messengers and regulate microneme secretion and proliferation in *T. gondii*. While PA mediated signaling is newly explored in protozoan parasites it is widely studied in other eukaryotes where its known to perform various cellular functions, including vesicular trafficking and secretion^7–10^. Given the regulatory role of PA, its production and metabolism is tightly controlled a type-2 Phosphatidic acid phosphatase (PAP2) that converts PA back to DAG.

Phosphatidic acid phosphatase (PAP) is known to catalyze dephosphorylation of PA to produce DAG and inorganic phosphate (P_i_). Brindley and co-workers were the first to describe existence of two types of PAP based on their substrate specificity and sub-cellular localization^11^. The soluble PAP named type-1 (PAP1) shows Mg^2+^-dependent activity and participates in triacylglycerol synthesis. On the other hand, the type-2 enzyme (PAP2) is plasma membrane-bound, Mg^2+^-independent and is involved in lipid mediated signal transduction and metabolism^12,13^. The first indication of regulatory role of PAP2 was seen in transformed fibroblasts cells where in decreased PAP2 activity led to imbalance of two secondary messengers, DAG and PA^14^. PA, the substrate of PAP2 is an active second messenger that is involved in a diverse range of processes such as vesicular secretion, endocytosis, recruitment of enzymes, etc. The action of PAP2 is, therefore, closely linked to the maintenance of PA homeostasis. Elevated or reduced levels of PA can induce stress like conditions leading to cell death in plant cell^15,16^.

In this study we identified a PAP2 homologue, PfPAP2 in *Plasmodium falciparum* and characterized its role in PA homeostasis. Conserved domain analysis shows that PfPAP2 contains the canonical acid phosphatase domain (APD) of PAP2 superfamily along with the conserved signature motifs. To characterize PfPAP2 as a typical PAP2 protein we cloned and expressed the predicted catalytic domain, i.e., the APD in *E. coli* and showed that it dephosphorylates PA. Inhibition of PAP activity of catalytic domain by propranolol was confirmed by LC-MS and other biochemical assays. Together these results corroborate the candidature of PfPAP2 as a member of the PAP2 superfamily with the role of a phosphatase in maintenance of PA homoeostasis.

Next, we demonstrate that accumulation of PA is lethal for the parasite with more than 90 % growth inhibition obtained in treated parasites. Upon further investigation into the plausible mechanism for this significant growth inhibition in propranolol treated parasites we found that blocking PfPAP2 activity causes egress of non-invasive merozoites that are impotent for progression of next cycle growth. The detailed analysis revealed untimely secretion of micronemes and perforin like protein 1 (PLP1) leading to permeabilization of host erythrocyte membrane facilitating the premature egress.

Our work highlights the role of PfPAP2 in maintaining PA homeostasis, which is essential for parasite to sustain and flourish in the host erythrocytes. This study presents PfPAP2 as a key player in maintaining PA homeostasis in malaria parasite, which can be exploited to develop an un-conventional drug development approach targeting PA homeostasis, which indeed is indispensable for parasite growth.

## Results

### Expression of a homologue of type-2 PAP2 in intra-erythrocytic life stages of *P. falciparum*

**Fig. 1:**
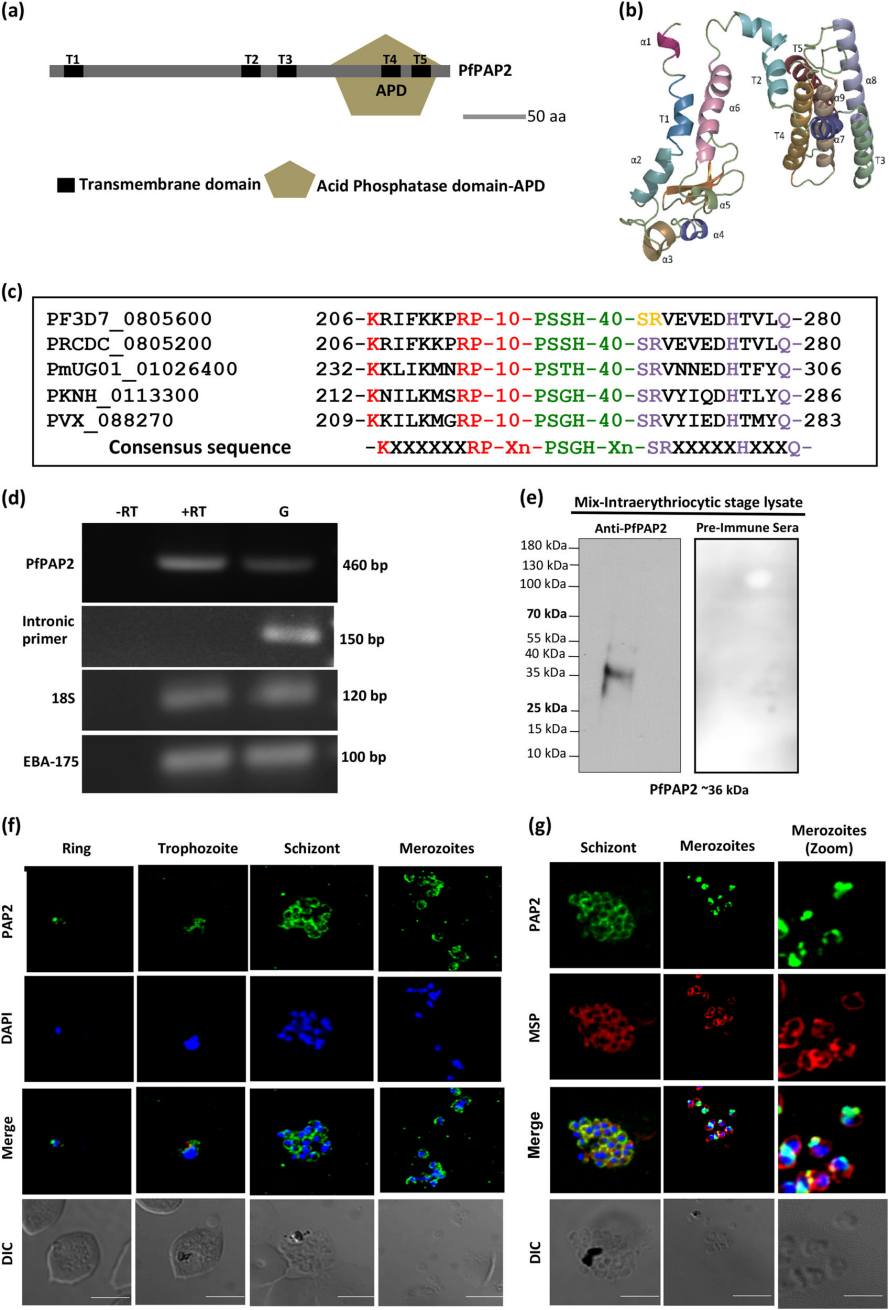
In silico analysis and expression of PAP2 homologue in malaria parasite, *Plasmodium falciparum*, PfPAP2. **a** Domain architecture of PfPAP2. PfPAP2 harbors all the canonical domains of PAP2 superfamily. It contains 5 trans-membrane domains marked in black (T1-T5) and an acid phosphatase domain (APD) highlighted in light green towards end of C-terminal. Acid phosphatase domain possesses the conserved signature acid phosphatase motif. **b** Homology model of PfPAP2 three-dimensional structure. Predicted 3D structure of PfPAP2 generated by Phyre^2^ server. The model was further processed for energy minimization and to reduce steric hindrances. APD lies between T2-T5, which are clustered together. **c** Characteristic signature motif of acid phosphatase domain (APD) of PAP2 superfamily is conserved across different species of *Plasmodium*. **K**XXXXXX**RP**-**PSGH**-**SR**XXXXX**H**XXXD is the characteristic signature motif of APD. Multiple sequence alignment of APD of PfPAP2 with APDs of other *Plasmodium* species shows that the consensus sequence is highly conserved across different *Plasmodium* species. **d** PfPAP2 transcript is present in the blood stage. An amplicon of 460 bp amplified using primer probes specific to PfPAP2 was detected in the cDNA isolated from blood stage parasite. Intron specific primers were used in RT-PCR analysis with cDNA as a negative control for genomic DNA contamination RNA samples isolated from unsynchronized blood stage parasites were reverse-transcribed (+RT) and used for detection of transcripts for PfPAP2, EBA175 and 18S rRNA by PCR using specific primers. These primers were also used for PCR with RNA without reverse transcription (−RT) to control for genomic DNA contamination and with *P. falciparum* genomic DNA (G) as a positive control. **e** Expression of PfPAP2 was also detected at protein level in *P. falciparum* merozoites by Western blotting. Mouse serum raised against rAPD detects a protein of ~36 kDa in merozoite lysates by Western blotting, corresponding to native PfPAP2. Pre-immune sera (PIS) did not recognize any proteins in the merozoite lysate. **f** Detection of PfPAP2 by IFA. **i** PfPAP2 is expressed throughout the erythrocytic schizogony. The localization of PfPAP2 was investigated in blood stages of *P. falciparum* by IFA using confocal microscope, Nikon A1. In the ring stage, PfPAP2 (green) was found around the nucleus stained with DAPI (blue). In trophozoites and late schizont stage, PfPAP2 (green) was found to give a honeycomb-staining pattern. **g** In the schizont and merozoite stage, colocalization of PfPAP2 (green) with PfMSP1 (red), a merozoite surface marker protein, revealed localization of PfPAP2 below cell membrane towards parasite cytosol

Using *Plasmodium* database (www.plasmoDB.org), we identified a gene, PF3D7_0805600 encoding for a putative PAP2 in *P. falciparum*. PAP2 superfamily members are characterized by the presence of multiple trans-membrane domains and an APD with conserved signature motifs (Fig. 1a). PfPAP2 sequence of 308 amino acids (aa) was analyzed with the online trans-membrane domain prediction software TMHMM and we found that PfPAP2 contains five putative trans-membrane domains- T1: 10aa-32aa, T2: 132aa-149aa, T3: 159aa-181aa, T4: 251aa- 268aa, T5: 278aa- 298aa. While searching for the catalytic APD using conserved domain database (CDD) we found that PfPAP2 possesses the characteristic acid phosphatase domain at the C terminal: 186aa-296aa. The APD of PfPAP2 superfamily harbors the conserved acid phosphatase signature sequence, KXXXXXXRP-Xn-PSGH-Xn-SRXXXXXHXXXQ, containing three motifs: C1, “KX_6_RP”, C2, “PSGH” and C3, “SRX_5_HX_3_D”^17–19^.

For structural characterization of PfPAP2 we generated a homology-based model using Phyre^2^ server in intensive mode (Fig. 1b)^20^. The crystal structure of *E. coli* phosphatidyl glycerophosphate phosphatase B, ecPgpB (PDB ID: 4PX7), a PAP2 superfamily member showed the maximum homology with PfPAP2 and was used as a template. The confidence score for the modeled PfPAP2 structure was 99.9% with a coverage area of 44%. The resultant 3D structure of PfPAP2 exhibits some similarity with folding topology of trans-membrane domains and catalytic domain as reported for ecPgpB^21^. The core helix bundle of PfPAP2 is formed by T2–T5, with T1 adjacent to the core. There are 9 alpha helices in the PfPAP2 structure. The putative active site formed by PfPAP2 signature motifs is located in the primary sequence from the C terminus of T3 to the N-terminal end of T5.

We performed sequence alignment with the APD domains of different species of *Plasmodium* and found that the characteristic consensus sequence is conserved across different species of *Plasmodium* (Fig. 1c) and as well as across all the other known members of PAP2 superfamily (Supplementary Fig. 1). To study the evolutionary relationship of PfPAP2 with other known members of PAP2 superfamily we constructed a phylogenetic tree using MEGA 7 software, which showed that PfPAP2 is evolutionary close to some members of PAP2 superfamily such as, ecPgpB, Chloroperoxidase of fungi, *Curvularia inaequalis*, and human Glucose-6-phosphatase and it is distantly related to human PAP2s (Supplementary Fig. 2)^17–19,22–24^.

To analyse the expression of PfPAP2 in blood stage at transcript level, semi-quantitative RT-PCR was performed. We detected an amplicon of 460 bp specific to PfPAP2, confirming the presence of PfPAP2 transcript (Fig. 1d). Intron specific primers were used in RT-PCR analysis with cDNA as a negative control for genomic DNA contamination. EBA-175 was used as late blood stage marker. 18S rRNA served as an internal control for housekeeping genes. The full agarose gel images for all individual genes are provided in the supplementary file as supplementary Fig. 3.

Antiserum against APD domain of PfPAP2 was generated in mouse, henceforth labeled as anti-PfPAP2. Expression of PfPAP2 in parasites at protein level was confirmed by Western blot analysis of parasite lysate containing majorly mature stages of *P. falciparum* using anti-PfPAP2 serum. A band of ~36 kDa in size corresponding to full length native PfPAP2 (predicted size, 35.85 kDa) confirmed the presence of PfPAP2 protein in *P. falciparum* (Fig. 1e). Pre-immune serum was used as negative control.

Immunofluorescence assays (IFAs) were performed using anti-PfPAP2 antibody to study the expression and localization of PfPAP2 at different asexual developmental stages of *P. falciparum*. PfPAP2 is expressed throughout the erythrocytic stages (Fig. 1f). In mature schizonts honeycomb like staining of PfPAP2 was observed. To further confirm the the staining of PfPAP2, anti-PfPAP2-peptide antibody was used. Anti-PfPAP2-peptide antibody was generated against a specific KLH conjugated peptide (aa_50_-aa_70_) of PfPAP2. The antibody against peptide also depicted similar localization in mature schizonts (Supplementary Fig. 4). Since, this kind of localization was reported for surface proteins of merozoites, we performed IFA in mature schizonts and merozoites using anti-PfPAP2 antibody and anti-MSP1 antibody. The MSP1 was found to localize to parasite surface both in merozoites and schizonts. PfPAP2 staining also showed similar arrangement around parasites. Although the staining looked similar, the low colocalization index suggests that it may be present towards the inner face of parasite cell membrane. (Fig. 1g).

### PfPAP2 dephosphorylates PA demonstrating the canonical PAP activity and can be blocked by an inhibitor of PAP2, propranolol

**Fig. 2:**
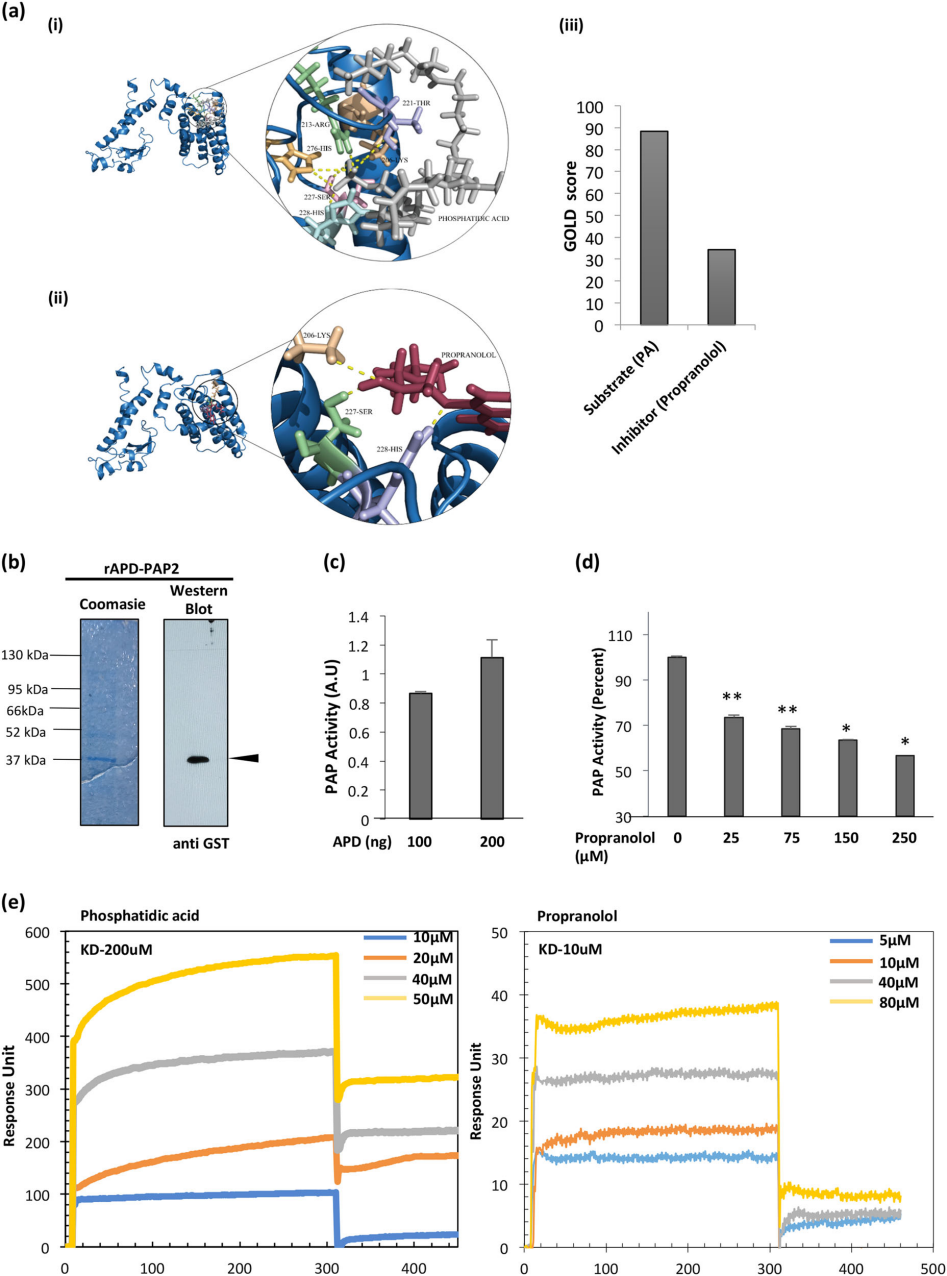
Propranolol, a canonical inhibitor of PAP2 inhibits dephosphorylation of PA by PfPAP2. **a** Active site prediction of PfPAP2 and docking with substrate PA and known PAP2 inhibitor, propranolol. Both ligands bind to the binding pocket with different affinity. (i) PA binds to the predicted active site that lies on APD with seven hydrogen bonds. (ii) Docking of known PAP2 inhibitor, propranolol against the active site present on APD (iii) GOLD score provides the quality of these docking represented in the form of bar graph. Substrate has a better score than inhibitor. **b** Expression of rAPD in the bacterial expression system. APD (~38.75 kDa) was eluted with reduced glutathione in Tris-NaCl buffer. Coomassie stained gels and corresponding Western blot analysis with anti-GST antibodies is shown confirming APD expression. A band corresponding to APD ~38.75 kDa was detected along with the degraded GST tag ~26 kDa in the eluted fractions. **c** Purified APD exhibited phosphatase activity. rAPD displayed phosphatase activity as suggested by malachite green assay. **d** Inhibition of APD activity by propranolol. Propranolol, its inhibitor, demonstrated dose-dependent inhibition of phosphatase activity of PfPAP2. *P < 0.05, **P < 0.01, (*n* = 3). **e** SPR analysis revealed binding of substrate, PA and inhibitor, propranolol to rAPD domain of PfPAP2. Dissociation kinetics of PA and propranolol was monitored with rAPD. PA demonstrated K_D_ value of 200uM while propranolol demonstrated K_D_ value of 10uM with rAPD domain

To get an insight into the binding pocket or active site of PfPAP2, we performed docking studies with the known PAP2 substrate, PA and inhibitor, propranolol against the active site present on APD that revealed that both these ligands bind to the same active site with different affinity for the predicted binding pocket present on the PfPAP2 catalytic domain, APD. The inhibitor, propranolol forms three hydrogen bonds with the PfPAP2 by interacting with three residues in the binding pocket (LYS-206, SER-227, HIS-228) whereas the substrate (Fig. 2a, i), PA forms seven hydrogen bonds by interacting with 6 residues (LYS-206, ARG-213, THR-211, SER-227, HIS-228, HIS-276) (Fig. 2a, ii). The docking score or the GOLD score of propranolol is 34.3, while the substrate docking score is 88.3 (Fig. 2a, iii). The substrate showed better GOLD fitness score compare to inhibitor, in consistence with the hydrogen bond analysis.

To functionally characterize PfPAP2, we cloned and expressed the catalytic acid phosphatase domain, APD (111aa long, 186aa-296 aa) in bacterial expression system as a GST fusion protein (Fig. 2b). Recombinant APD, rAPD (12.75 kDa) was detected at the expected size of ~38.75 kDa (12.75 kDa-APD+26 kDa-GST) by Western blotting using anti-GST antibody (Fig. 2b). PAP2 dephosphorylates PA to yield DAG and P_i_. The activity of rAPD was measured by quantifying the amount of _Pi_ released using a Malachite Green based assay (Fig. 2c). In this assay, released P_i_ forms a complex with Molybdate ions and Malachite Green dye and gives a green color complex that can be measured using spectrometer. The assay demonstrated that rAPD dephosphorylated PA and this activity could be blocked by propranolol in dose-dependent manner (Fig. 2d).

Surface plasmon resonance (SPR) can be used to analyze binding kinetics between two molecules. It gives detailed insight into protein-ligand and protein-drug interactions. To confirm that PfPAP2 is PA phosphatase and propranolol is its canonical inhibitor we performed SPR analysis of PfPAP2 with PA and propranolol. We demonstrate that PA, can bind to PfPAP2 with a K_D_ value of 200 μM while propranolol has a much lower K_D_ value of 10 μM suggesting that propranolol has a very high affinity for PfPAP2 (Fig. 2e).

### Inhibition of PfPAP2 activity by propranolol disrupts PA homeostasis

**Fig. 3:**
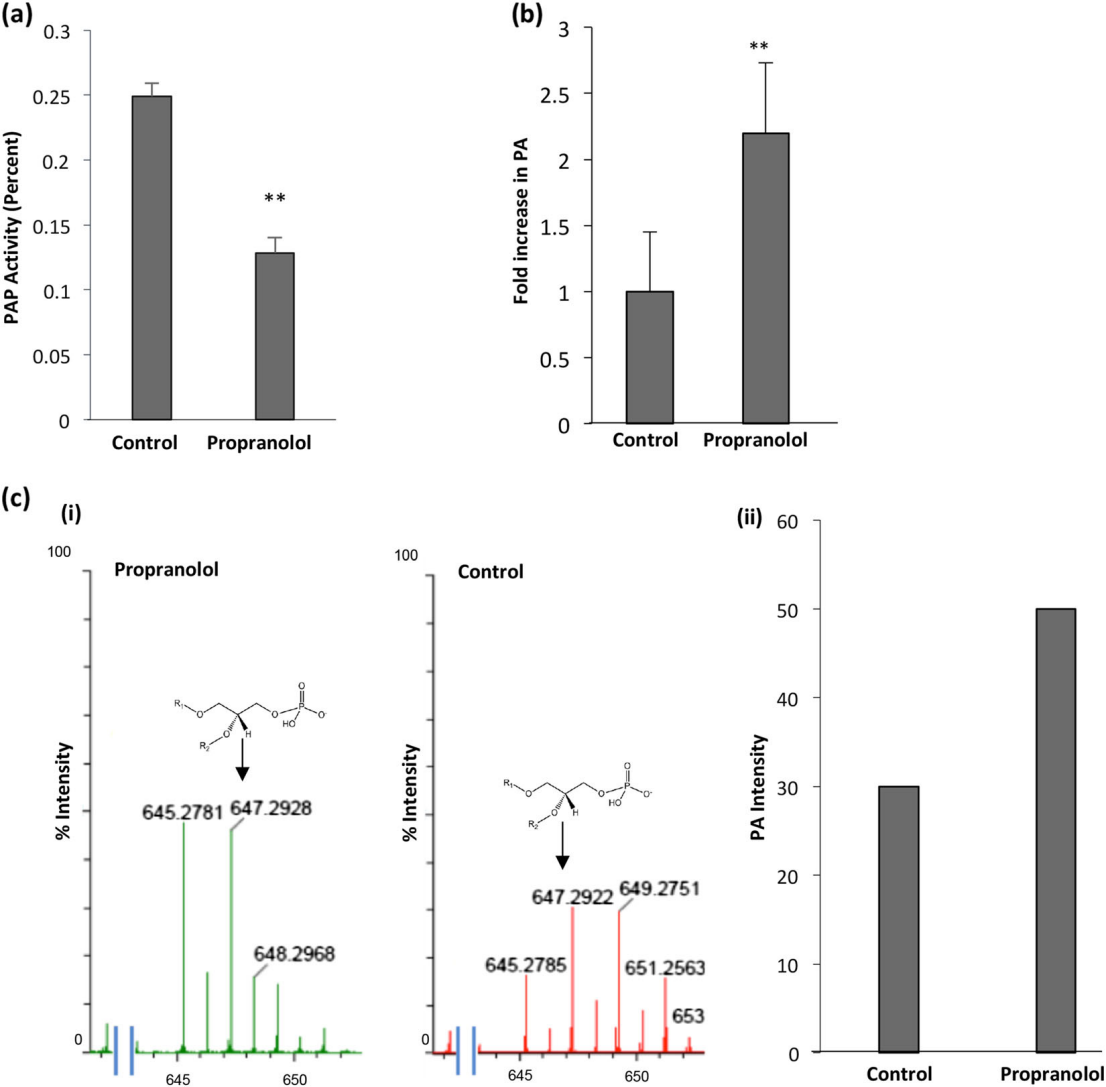
PAP activity and accumulation of PA exhibited by PfPAP2 in malaria parasite. **a** Lysate of mature blood stages of malaria parasites demonstrated PA dependent phosphatase activity. Lysate of mature blood stages of parasite release Pi in the presence of PA which is inhibited by propranolol (125uM) indicating activity of PfPAP2. **P < 0.01, (*n* = 3). **b** Lysate of mature blood stages of malaria parasites accumulates PA in the presence of propranolol (125 μM). **P < 0.01, (*n* = 2). **c** (i) PA accumulation in parasite lysate in the presence of propranolol (125 μM) was monitored by LC-MS which demonstrated higher intensity peak of 647.29 *m/z* that corresponds to PA. (ii) Quantification of LC-MS peak intensity establishes accumulation of PA in response to propranolol

PAP2 is responsible for maintaining the intracellular levels of PA and any kind of imbalance may lead to deleterious effects on growth and survival of parasite. To confirm the presence of PA dependent phosphatase activity in malaria parasites, we analyzed the release of Pi in the presence and absence of propranolol in lysate of mature blood stages. We found the presence of phosphatase activity in parasite lysate which is decreased following addition of propranolol (Fig. 3a, Supplementary Fig. 5).

We, further, blocked the activity of PfPAP2 with propranolol and measured the amount of PA in propranolol treated and untreated parasites using fluorimetry-based assay. We observed a 1.5-fold increase in PA in parasites treated with propranolol resulting in PA imbalance in *P. falciparum* (Fig. 3b). We demonstrate that propranolol inhibited the parasite specific PA dependent phosphatase activity leading to accumulation of PA.

To further confirm the accumulation of PA in the parasites, mass spectrometric analysis was performed. We could detect peak corresponding to parent ion of PA, *m/z* 647.29, both in the control and propranolol treated samples (Fig. 3c, i). Quantitation of the peak intensity revealed accumulation of PA in the propranolol treated parasites (Fig. 3c, ii). This data confirms that parasites express a functional PAP to tightly regulate the intracellular levels of PA.

### Inhibition of PfPAP2 activity expedites preterm microneme secretion, host cell membrane permeabilization facilitating premature egress

**Fig. 4:**
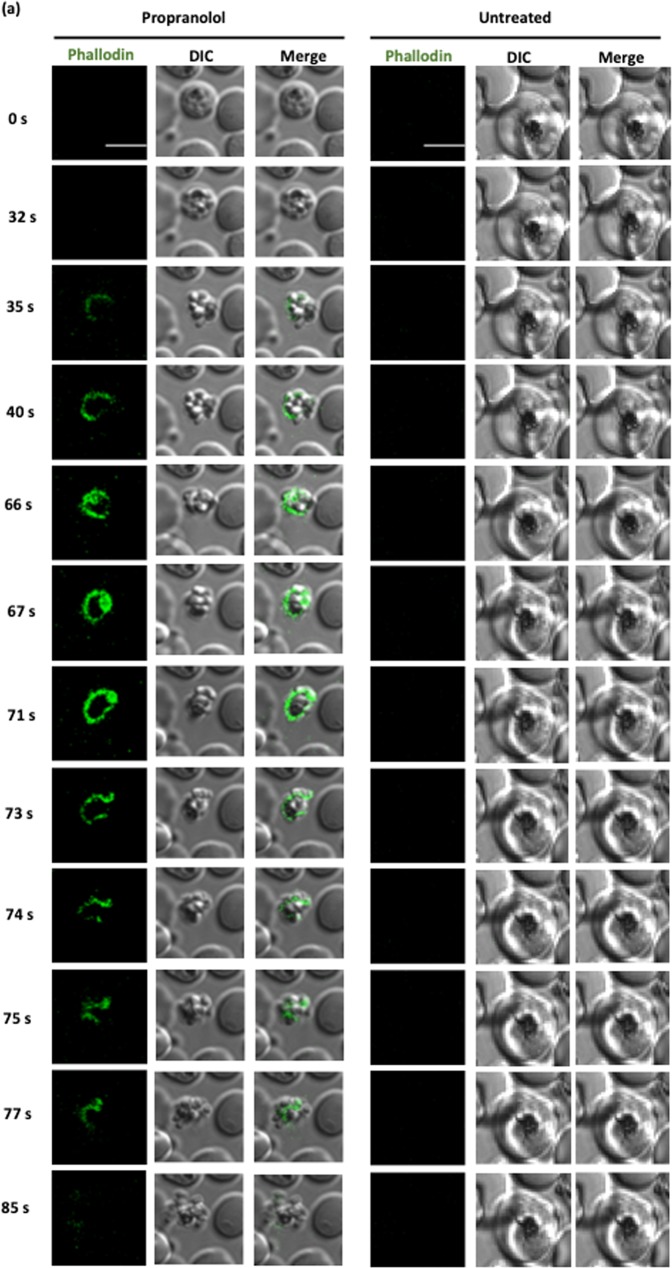

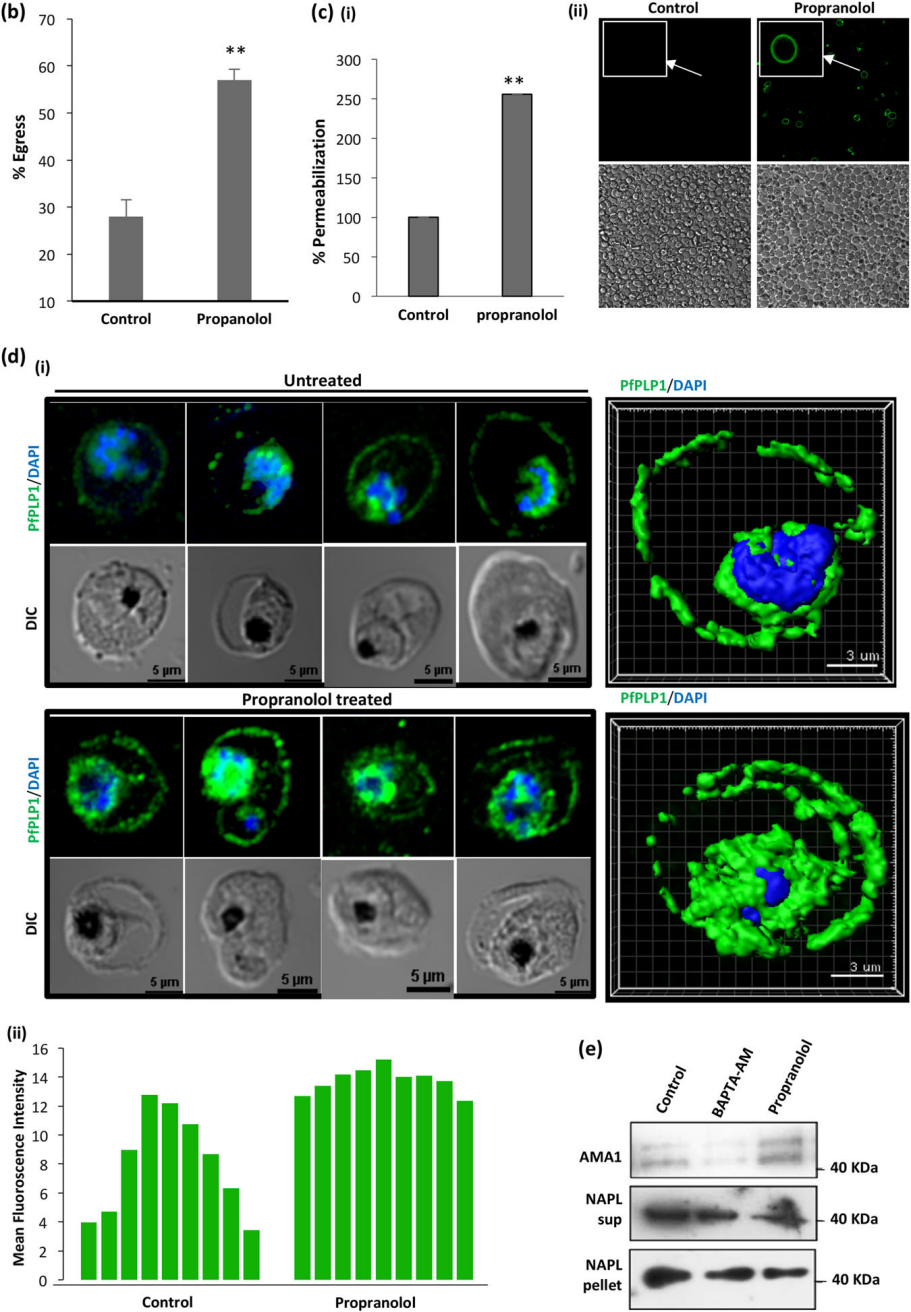
Accumulation of PA due to propranolol mediated inhibition of PfPAP2 induces premature microneme secretion, host RBC membrane permeabilization leading to preterm egress. **a** Merozoite egress and host cell membrane permeabilization was monitored by time-lapse microscopy in the presence of 100 μM Phalloidin Alexa 488. Host cell membrane permeabilization accompanied by egress of merozoites was observed following propranolol (125 μM) treatment of parasites while untreated parasites did not demonstrate preterm egress. **b** Egress of merozoites was quantified using Giemsa staining and visualized under microscope. The counting data demonstrated that propranolol (125 μM) treatment induced approx. two-fold egress as compared to control. **P < 0.01, (*n* = 3). (**c**) Host cell membrane permeabilization in the presence of propranolol (125 μM). (i) Percent of phalloidin positive schizonts, demonstrating host erythrocyte membrane permeabilization, increased in the presence of propranolol. **P < 0.01, (*n* = 2) (ii) Corresponding confocal images of the treated and untreated schizonts in the presence of Phalloidin Alexa 488. **d** Localization of PLP1 on host erythrocyte membrane. (i) Propranolol (125 μM) treated and untreated schizonts were stained with anti-PLP1 antibody and visualized under confocal microscope. 3-D reconstruction of the confocal images by IMARIS is also shown. PLP1 is localized more on host erythrocyte membrane as compared to untreated control. (ii) To score the increased localization of PLP1 on membrane, we created ROI specific to membrane of RBC and analyzed intensity of PLP1 staining. The results demonstrate that propranolol treated schizonts have higher intensity of PLP1 on membrane as compared to control. **e** Microneme secretion is triggered in the presence of propranolol (125 μM). Merozoites were isolated and incubated in the presence and absence of BAPTA-AM and propranolol. Microneme secretion was assessed in the merozoite supernatant. Enhanced AMA1, a micronemal marker, secretion was observed in supernatant of propranolol while BAPTA-AM was used as a negative control. NAPL worked as loading and lysis control

To understand the role of PA in malaria parasites, we triggered accumulation of PA using propranolol and analyzed the egress kinetics. We found that propranolol treatment is inducing egress of merozoites before time when compared to untreated control (Fig. 4a, Supplementary Video 1, Supplementary Video 2, Supplementary Video 3 and Supplementary Video 4). Further, detailed analysis of egress kinetics was performed using time-lapse microscopy wherein we found that propranolol treatment is inducing preterm egress as compared to control following host cell membrane permeabilization (Fig. 4a, b, Supplementary Video 1, Supplementary Video 2, Supplementary Video 3 and Supplementary Video 4). Quantification of propranolol treated schizonts revealed more then two-fold increase in host cell permeabilization as compared to control (Fig. 4c, i, ii).

Perforin like proteins (PLP) of malaria parasites have role in host cell membrane permeabilization to facilitate egress^25,26^. In the blood stage two PLPs are expressed, PLP1 and PLP2, that play role in merozoite egress. Expression of two PLPs in the same stage for similar tasks is suggestive of their redundancy in *P. falciparum*^26^. The localization of PLP1 serves as a fingerprint for micronemal discharge and enhanced permeabilization.

In the presence of propranolol, the egress is facilitated, therefore it is difficult capture schizonts and score PLP1 localization. To overcome this limitation, we pretreated schizonts with a protease inhibitor, E-64, limits egress of parasites but not obstruct the localization of PLP1. The pretreated schizonts were then scored for PLP1 localization in the presence or absence of propranolol. We found that PLP1 is localized more to host RBC membrane in PA elevated schizonts as compared to control (Fig. 4d, i). Mean fluorescence intensity of schizonts further confirmed higher membrane localization of PLP1 in propranolol treated schizonts (Fig. 4d, ii). Since, PLP1 is a micronemal protein, its localization on membrane is governed by various triggers of microneme secretion. To access PA accumulation is triggering micronemal discharge in malaria parasites free merozoites were treated with or without propranolol and BAPTA-AM and secretion of a micronemal marker was evaluated using Western Blot (Fig. 4e, Supplementary Fig. 6). AMA1 secretion was triggered by propranolol while BAPTA-AM inhibited its secretion.

### Propranolol mediated PfPAP2 inhibition restricts parasite growth due to abortive invasion

**Fig. 5:**
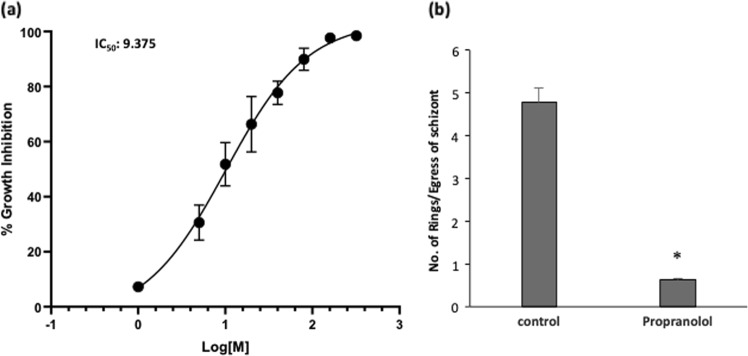
Inhibition of PfPAP2 activity restricts parasite growth. **a** Inhibition of *P. falciparum* intra-erythrocytic growth. Troph stage parasites were treated with different concentrations (1–500 µM) of propranolol over one complete intra-erythrocytic life cycle of the parasite. Growth inhibition was measured using SYBR green staining. Propranolol inhibited parasite growth in a dose-dependent manner. **b** Reduced ring formation in propranolol treated parasites. Number of rings formed per schizont egress was calculated to score invasion. Number of invasion (rings formed) per schizont egress was highly reduced in propranolol treatment. *P < 0.05, (*n* = 3)

To evaluate the effect of PfPAP2 inhibition on parasite growth we treated parasite at trophozoite stage with increasing concentration of propranolol (1–400 μM) and allowed them to grow for one cycle. The growth rate of parasite was evaluated using SYBR Green. We found that propranolol inhibited parasite growth in a dose-dependent manner (1–400 μM) (Fig. 5a). The IC_50_ was calculated and found to be 9.375 μM.

**Fig. 6:**
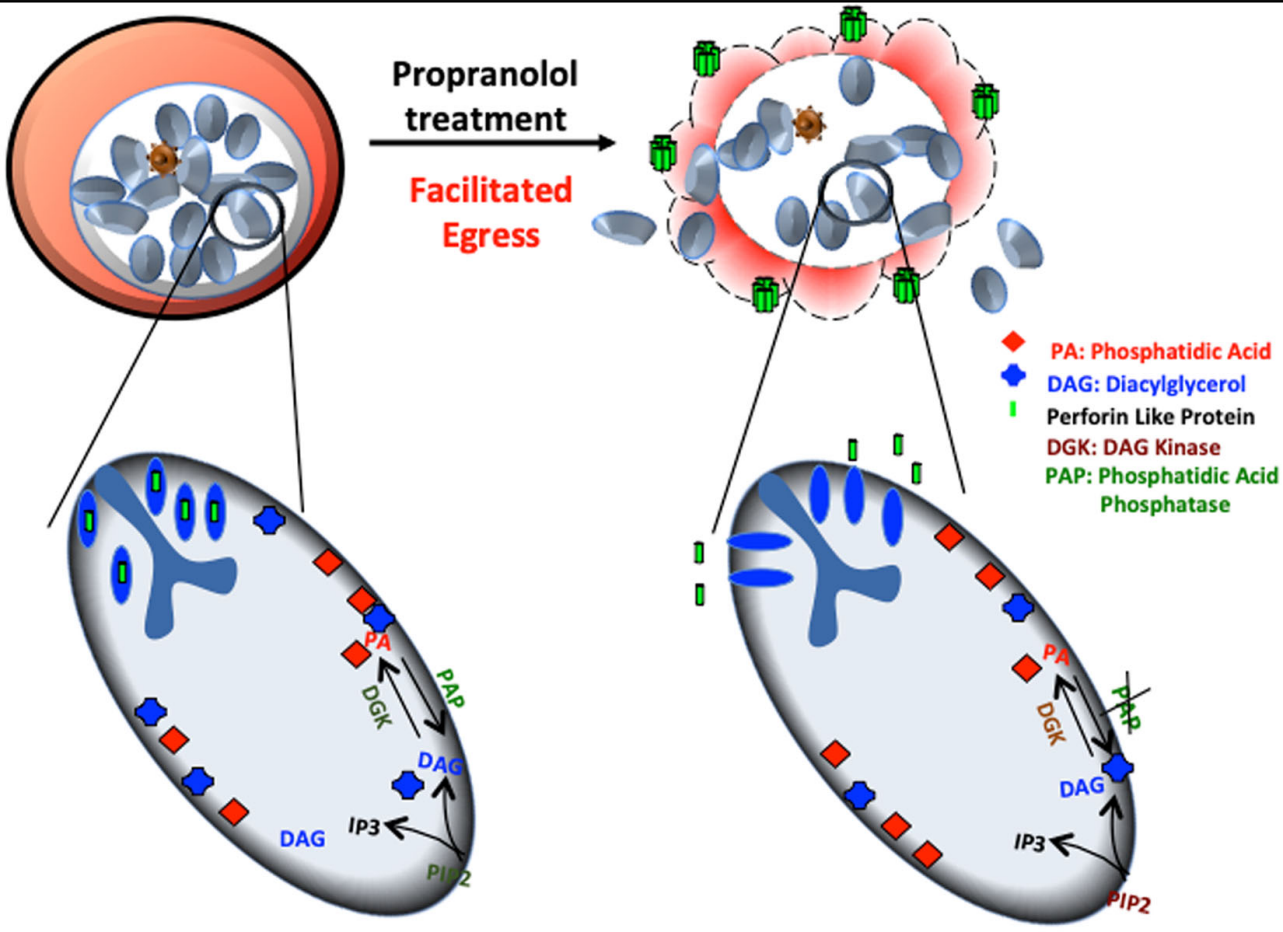
Model for regulation of PA homeostasis by PfPAP2 in malaria parasite. PA homeostasis maintained by PfPAP2 is essential for parasite growth. Blocking the PAP activity of PfPAP2 creates PA imbalance in *P. falciparum*. This breakdown in PA homeostasis promotes preterm egress of merozoites, which then fail to survive in the host environment leading to limited growth of parasite. PfPAP2 clearly stands out as an important regulator, maintaining the PA homeostasis that is crucial for parasite to flourish

To dissect the mechanism of growth inhibition (Fig. 6), we presumed that premature egress of merozoites is making them incompetent to invade. Since forced and premature egress of merozoites was observed, we devised a novel parameter to score invasion, which can eliminate the inadvertent errors of egress in invasion. Herein, we calculated number of newly rings formed per successful egress of schizonts (Fig. 5b). We found that efficiency of propranolol treated merozoite invasion is highly reduced. Thus, we confirm that disruption of PA homeostasis due to inhibition of PfPAP2 by propranolol led to restricted growth rate of *P. falciparum* due to a cumulative effect of preterm egress and abortive invasion.

## Discussion

Understanding of lipid mediated signaling and its role in the malaria parasite, *Plasmodium* is in a very primitive stage unlike other eukaryotes where the roles of lipid second messengers are well documented. PA, a key lipid messengers is involved in various processes such as endocytosis, vesicular secretion, membrane trafficking, etc.^7–10^. Homeostasis of PA is very crucial given the wide range of processes it facilitate and regulate. One of the key enzymes that maintain balance this balance is PAP2, which dephosphorylates PA to yield its substrate, DAG and _Pi_. Until now PAP2 and its functions have been reported in many eukaryotes but not much is known about its presence and role in *Plasmodium*^11–13,27^.

Given the function of PAP2 in maintaining PA homeostasis we were intrigued to identify and study the homologue of PAP2 in *P. falciparum* if any. To our interest, we found a gene, Pf3D7_0805600 in the *Plasmodium* database, PlasmoDB encoding for a putative PAP2 and we named it as PfPAP2. PfPAP2 is a multi-trans-membrane domains containing protein with an APD at C-terminal, both salient features of members of PAP2 superfamily (Fig. 1a). PfPAP2 also possess the conserved signature motifs of catalytic domain: C1, “KX_6_RP”; C2, “PSGH”; and C3, “SRX_5_HX_3_D”^17–19^. PfPAP2 shares structural homology with bacterial PAP2 superfamily member, *E.coli* phosphatidyl glycerophosphate phosphatase B, ecPgpB^19^. The five trans-membrane domains of PfPAP2 are arranged in a similar pattern like that of ecPgpB where in the active site is present on the catalytic domain located between T3-T5 towards the periplasmic side (Fig. 1b)^21^.

A transcript of size 460 bp obtained by RT-PCR confirmed the presence of PfPAP2 in parasite cDNA (Fig. 1d). We detected a band of ~36 kDa corresponding to the native PfPAP2 of 35.75 kDa (predicted) in membrane fraction of parasite lysate using a highly specific antibody against the APD domain of PfPAP2 confirming the expression of PfPAP2 in *Plasmodium* (Fig. 1e). We used anti-PfPAP2 antibody in immunofluorescence experiments to study the localization of PfPAP2 in the asexual stage of *P. falciparum*. We found PfPAP2 expression throughout the asexual stage of *P. falciparum* but majorly in the schizonts and merozoite stages of the parasite (Fig. 1f). Using PfMSP-1, as a membrane marker, we show that staining pattern of PfPAP2 is similar to PfMSP1 but it may be located below the parasite membrane towards the parasite cytosol (Fig. 1g). These results confirm the presence and expression of a PAP2 homologue in *P. falciparum*.

PAP2 proteins can dephosphorylate a wide range of substrates including PA^11–13,28^. PA is dephosphorylated by PAP2 to produce DAG and _Pi_. Heterologous expression of its catalytic domain, APD (111 aa) in mammalian and bacterial expression system respectively yielded us the GST tagged APD ~38.75 kDa (Fig. 2b). rAPD exhibited PAP activity, which was inhibited by the known inhibitor of PAP2, propranolol (Fig. 2d). Propranolol has been previously reported to inhibit PAP2 activity in other systems^28^. Inhibition of PAP activities of rPfPAP2 and APD by propranolol, therefore suggest that PfPAP2 demonstrates characteristic PAP activity exhibited by other PAP2 superfamily members. In silico studies substantiated our in vitro data. Docking studies with PAP2 substrate, PA and its inhibitor, propranolol against the homology modeled PfPAP2 showed that both PA and propranolol bind to the same active site on the predicted catalytic domain of PfPAP2 in consistence with the inhibition obtained with propranolol (Fig. 2a, i and ii). Further, SPR analysis confirmed the binding of PA and propranolol to the APD domain of PfPAP2 (Fig. 2e).

A recent study in apicomplexan member, *Toxoplasma gondii*, showed that PA mediates microneme secretion and ultimately the process of invasion and egress in *T. gondii*^6^. Since PA homeostasis is necessary to mediate key processes required for parasite survival and growth in the host, any kind of imbalance may impose serious detrimental effect on the parasite. We disrupted PA homeostasis by inhibiting activity of PfPAP2 using propranolol (Fig. 3a). Accumulated PA was quantified using a fluorimetry-based method and we observed a 1.5-fold increase in propranolol treated parasite compared to control (Fig. 3b). The accumulation of PA was validated by mass spectrometery that also corroborated our findings (Fig. 3c, i). This PA imbalance created in *Plasmodium* restricted the growth of parasite as observed by inhibition of parasite growth in a dose-dependent manner when treated with propranolol (Fig. 5a). To dwell deeper into the mechanism that limits parasite growth we investigated the effect of propranolol mediated PfPAP2 inhibition on egress of merozoites in *P. falciparum*. We observed that blocking PAP activity of PfPAP2 leads to premature egress of *P. falciparum* merozoites. Further dissection of this propranolol mediated expedited egress revealed that PfPAP2 inhibition triggered micronemal secretion of PfPLP1 to host erythrocyte membrane, facilitating its permeabilization and egress (Fig. 4, Supplementary Video 1, Supplementary Video 2, Supplementary Video 3 and Supplementary Video 4). This stimulated egress of under-prepared parasites caused by inhibition of PfPAP2 activity is responsible for decreased efficiency of merozoites to form rings and hence majorly responsible for limiting parasite growth (Fig. 5).

To conclude, we confirm the presence and expression of PAP2 homologue in *Plasmodium* and establish its role as a regulatory enzyme, which is attributed to its contribution in maintaining the PA homeostasis. We show that inhibiting the PAP activity of PfPAP2 causes accumulation of its substrate, PA, which limits the growth of parasite by facilitating the forced exit of merozoites into the host premises (Fig. 5). When turned off it can pose a serious threat to the malaria parasite, limiting its growth that eventually culminates in death of this deadly parasite. Our study presents a new candidate for antimalarial drug designing i.e. PfPAP2, which acts a negative feedback switch in PA mediated signaling. Furthermore, structural and phylogenetic analysis (Fig. 1 and Supplementary Figs. 1, 2) suggests that PfPAP2 is very close to its bacterial counterpart, ecPgpB while distantly related to human PAP2s, thus, PfPAP2 emerges as a unique and a potential target for antimalarial drug discovery.

## Materials and methods

### In vitro culture of *P. falciparum*

Frozen stocks of *P. falciparum* 3D7 strain were thawed and cultured using a modified Trager and Jensen method^29,30^. Parasites were maintained in complete RPMI (RPMI 1640, Invitrogen, USA), supplemented with 27.2 mg/L hypoxanthine (Sigma, USA), 0.5 gm/L Albumax I (Gibco, USA) and 2 gm/L sodium bicarbonate (Sigma, USA) and O^+^ human erythrocytes, under mixed gas conditions (5% O_2_, 5% CO_2_ and 90% N_2_) at 37 °C.

### Bioinformatics analysis

The sequence of *P. falciparum* type-2 Phosphatidic acid phosphatase, PAP2 (PfPAP2: PF3D7_0805600) was retrieved from PlasmoDB database (http://plasmodb.org). Domains of PfPAP2 were identified using the CDD available at NCBI (www.ncbi.nlm.nih.gov/structure/cdd /wrpsb.cgi). Multiple sequence alignment of PfPAP2 sequence was performed using Clustal OMEGA^30–32^. Phylogenetic tree was constructed based on maximum likelihood method using online software, MEGA version 7^31^. TMHMM server v.2.0 was to predict the trans-membrane helices in PfPAP2^32^.

### Protein structure prediction

The 3D structure for PfPAP2 is homology modeled using Phyre^2^ V 2.0 (Protein Homology/analogy Recognition Engine) server available online^20^. The models were screened for unfavorable steric contacts and remodeled using either a rotamer library database of SCHRODINGER.3 Explicit hydrogens were added to the protein and were subjected to energy minimization using OPLS2005 force field in SCHRODINGER. Energy minimization and relaxation of the loop regions was performed using 500 iterations in a simple minimization method. The 3D energy minimized model evaluation was performed in PROCHECK^33^. The quality of modeled structure was validated by Ramachandran plot.

### Ligand-binding site prediction

The modeled structure of PfPAP2 protein was analyzed for potential binding cavities using the MetaPocket 2.0 server^34–36^. MetaPocket2.0 server was used to identify ligand-binding sites on the protein surface.

### Docking studies

The structures of PAP2 substrate Phosphatidic acid (1,2-Dioleoyl-sn-glycero-3-phosphoric acid sodium salt (CID:16212747), and PAP2 inhibitor, Propranolol (CID:4946) were retrieved from PubChem database. The 2D-structure data file (SDF) of compounds was converted into a 3D-MOL2 file with the program openbabel. In pre-docking preparations, hydrogens were added followed by minimization and optimization in an OPLS_2005 force field. Finally, ten confirmations for each ligand were generated and used for docking. Docking studies were performed using GOLD software^36^. The standard default setting is used to dock the ligands with PfPAP2 in GOLD.

### Reverse transcript analysis of PfPAP2 protein

RNA was isolated from mixed asexual stage saponin lysed *P. falciparum* using RNeasy mini-columns (Qiagen, Germany) according to the manufacturer’s instructions. RNA (~ 500 ng) was used for complementary DNA (cDNA) preparation using SuperScript II kit (Invitrogen, USA). cDNA was used as template to detect transcripts for PfPAP2, EBA175, Intronic region and 18S rRNA with gene specific oligonucleotide primers and Taq DNA polymerase (Fermentas, USA) by polymerase chain reaction (PCR). Sequences for oligonucleotide primers used for PCR for PfPAP2, EBA175, Intronic region and 18S rRNA were as follows: PfPAP2 Fwd: 5′-GTAGTAAAAAGATAAATAGTTTGA 3′, PfPAP2 Rev: 5′-GGCAATGCA CTATTAATTGGT3′, EBA-175 Fwd: 5′-AATTTCTGTAAAATATTGTGACCATATG-3′, EBA-175 Rev: 5′-ATACTGCACAACACAGATTTCTTG-3′, 18SrRNA Fwd: 5′-CCGCCCGTCGCTCCTACCG-3′, 18SrRNA Rev: 5′-CCTTGTTACGACTTCTCCTTCC-3′, Intronic region Fwd: 5′-GTTGATTAACAGTATGAGGTGATACATCCC-3′, Intronic region Rev: 5′-CGTTGTTACGACTTCGCCTTCC- 3′. The PCR products were analyzed by agarose gel electrophoresis to confirm the amplicon size.

### Heterologous expression of PfPAP2 APD in bacterial expression system

The 336 bp APD was amplified using primers: Fwd 5′ GTGCGGATCCAAAAACTTACTTTATATTATTTTTATAATGCCG 3′ and Rev 5’ATATGTCGACATAGAAAATAAATCCAAAACCTAT 3’and cloned in pGEX-4T-1 vector (GE Healthcare) at BamHI and SalI sites and used for expression in *E. coli* SHuffle 30. The resultant clone is labeled as APD-PfPAP2. Expression of recombinant APD-PfPAP2 was induced at 0.6-0.8 OD (600 nm) with 1 mM IPTG at 37 °C for 5 h. The cell biomass was resuspended in the lysis buffer containing 1 mM EDTA, 25 μg/ml lysozyme, 3 mM βME and protease inhibitor cocktail (Roche) in PBS. Lysate was loaded on 1 ml GSTrap FF prepacked columns (GE Healthcare). Recombinant APD-PfPAP2 was eluted with 10 mM and 20 mM reduced glutathione in 50 mM Tris (pH 8.0) and 50 mM NaCl. The final protein was buffer exchanged with Tris-NaCl buffer to remove glutathione and other impurities using a 10 kDa cut off Amicon filters (Millipore Inc., USA). Puriifed APD was evaluated for its PAP activity. The coomassie stained SDS gels were scanned using Fluor^TM^Chem M system (Protein simple, USA) in gray scale.

### Generation of PfPAP2 specific antisera

rAPD-PfPAP2 was used for generating polyclonal sera in mice. Five to six-week-old female BALB/c mice were immunized with 25 μg of purified rAPD-PfPAP2 protein formulated with complete Freund’s adjuvant. Animals were boosted twice with 25 μg of corresponding immunogen formulated with Freund’s incomplete adjuvant at 3 weeks interval. The sera was labeled as anti-PfPAP2 antibody.

A 20 amino acid peptide (aa_50_-aa_70_) with highest B-cell epitope score, predicted using online server, BepiPred-2.0, was selected and synthesized commercially conjugated to keyhole limpet hemocyanin (KLH) at C-terminal. Specific sera against this peptide was generated in mouse and labeled as anti-PfPAP2-peptide antibody.

### Immunoblot analysis of PfPAP2

For immunodetection of PfPAP2, we generated antibody in mice against rAPD domain. We labeled this antibody as anti-PfPAP2 antibody. PfPAP2 is an integral membrane protein so we isolated the membrane fraction of parasite using RIPA buffer and then solubilized it with 1% w/v of β-octylglucopyranoside (Sigma, USA). Proteins were separated by SDS-PAGE, transferred to nitrocellulose membrane using Trans-Blot SD Semi-Dry Transfer Cell (Bio-Rad, USA). The membrane was probed with anti-PfPAP2 mouse sera at a dilution of 1:200 and developed using ECL (enhanced chemiluminescence) Plus Western Blotting Detection system kit (GE Healthcare, USA) following the manufacturer’s specifications. Pre-immune sera were used as negative control.

### Immunofluorescence microscopy

Synchronized *P. falciparum* culture at each stage, i.e., rings, trophozoites schizonts and merozoites was smeared on glass slides, dried and fixed with pre-chilled methanol. Fixed smears were probed with anti-PfPAP2 mouse sera (1:50 dilution) and anti-PfMSP1 rabbit sera (1:100). After washing, smears were incubated with Alexa-Fluor 488-conjugated goat anti-mouse IgG (1:200, Molecular Probes, USA) and Alexa-Fluor 594 conjugated goat anti-rabbit IgG (1:500 dilution, Molecular Probes, USA) for 1 h at RT. The slides were washed, mounted with GOLD anti-Fade DAPI (Molecular Probes, USA) and analyzed using a Nikon A1 confocal microscope. NIS-elements software was used for processing of images.

Methanol-fixed slides of schizont stages of *P. falciparum*-infected RBC treated with propranolol were blocked with 3% BSA in PBS for 2 h at room temperature (RT) and probed with anti-PfPLP1 mouse sera diluted 1:100, followed by Alexa-Fluor 488-conjugated goat anti-mouse IgG antibody, diluted 1:200 for 1 h at RT. The slides were washed and mounted with DAPI antifade mounting media (Molecular Probe, Eugene, OR, USA).

### Determination of PAP activity of APD of PfPAP2

PAP dephosphorylates PA to generate diacylglycerol (DAG) and P_i_. To assess the activity of full length PfPAP2 and its catalytic domain, amount of P_i_ released was quantitated using Malachite Green assay kit (Bioassays systems, USA). Malachite green based estimation of PAP activity is already used for yeast system^37^. In this assay, molybdate ions along with Malachite Green forms a green color complex with P_i_ released from the phosphatase reaction and can be measured spectrophometrically at wavelength 650 nm. The reaction buffer comprises of 100 mM Tris pH 7.4 and 1 mM DTT. PA (Enzo Life Sciences, USA) was used as a substrate in the reaction. To initiate the phosphatase reaction, PfPAP2 or APD was incubated with reaction buffer and propranolol for 1 h at 30 °C. PA at final concentration of 125 μM and 250 μM was added to the reaction mixture and incubated for 1 h at 30 °C. The reaction is stopped by adding the detection reagent mixture and read at 650 nm wavelength to quantify the amount of P_i_ released. A standard curve is plotted and the concentration of PA is calculated within samples using the standard curve (Supplementary Fig. 7a).

### Surface plasmon resonance (SPR)

To determine the interactions of lipid PA and inhibitor of PAP2 Propanolol, SPR was carried out by using Auto Lab Esprit SPR. Affinity constant, K_D_ were measured at room temperature.10 μM of recombinant APD protein was immobilized on the surface of the sensor chip. Interaction analysis was studied by injecting Propranolol at different concentrations: 80, 40, 10, and 5 μM and Phosphatidic acid 50, 40, 20, and 10 μM over the chip surface, with association and dissociation time of 300 and 150 s, respectively. PBS buffer was used both as immobilization and binding solutions. Surface of the sensor chip was then regenerated with 50 mM NaOH solution. Data were fit by using Auto Lab SPR Kinetic Evaluation software provided with the instrument.

### Total PA extraction and estimation in *P. falciparum* by fluorimetric based method

We measured total PA content in propranolol treated and untreated parasite using Total Phosphatidic Acid Assay Kit, Cell Biolabs, Inc., USA. This kit is based on a coupled enzymatic reaction system, where, firstly a lipase is used to hydrolyze PA in samples to yield glycerol-3-phosphate (G-3-P), followed by its oxidation by G-3-P oxidase to produce hydrogen peroxide, which then reacts with the kit’s fluorometric probe. The final fluorescent product is estimated at excitation wavelength 530-560 nm and emission wavelength 585–595 nm. To begin, percoll purified schizonts were treated with propranolol at 125 μM concentration and incubated at 37 °C for 6 h. After treatment, PA was extracted and estimated in harvested parasite pellet using manufacturer’s instructions. Briefly, parasites were lysed by sonication with a pulse of 5 sec ON and 5 s OFF for 2 min (Q500, Q Sonicator, USA) and then mixed with methanol, NaCl and chloroform in the ratio of 1.5:1:2.5. After centrifugation, the upper aqueous phase is discarded and the lower chloroform phase is washed twice with pre-equilibrated upper phase (PEU) prepared by mixing chloroform and methanol in 1:1 ratio along with NaCl. Finally the lower organic phase is dried in a speed-vac (Concentrator plus, Eppendroff, USA). The dried sample is resuspended in 50 μl of 1X assay buffer. PA standards along with parasite samples were added to a 96 well micro-titer plate and proceeded for PA estimation. A standard curve is plotted and the concentration of PA is calculated within samples by comparing the sample fluorescence to the standard curve (Supplementary Fig. 7b). The amount of PA is normalized with the protein content in the parasite lysate estimated using BCA protein estimation kit (Pierce, USA). The fold increase in PA content per μg of parasite lysate is plotted in a bar graph.

### PA detection by mass spectrometry

Pellets were extracted following a previously described protocol^38^. PA level was measured in propranolol treated and untreated parasite pellets were diluted with 100 µL water, To each 100 mL aliquot 1.33 mL of 3 M KCl and 0.5 mL methanol were added. Samples were vortexed and sonicated to homogeneity, and then extracted with 2 mL of isopropanol/hexanes (1:2). The top layer was collected in glass tube and the bottom layer was re-extracted with 2 mL isopropanol/hexanes. The combined organic layers were dried in nitrogen gas and resuspended in 500 µL hexanes, dried, resuspended in 300 µL methanol, dried, and resuspended in 200 µL chloroform for LC-MS analysis.

Extracted lipids sample were subjected to LC-MS analysis. We used a Waters Acquity H-Class UPLC-system (Waters, Milford, MA, USA). Chromatographic separation was achieved on an Acquity BEH C18, 1.7 μm, 75 × 2.1 mm column (Waters, Manchester, UK). The mobile phase consisted of 0.1% Formic Acid Solvent (A) and acetonitrile (B). The initial gradient condition was 90% A and 10%B, 80% and 20% for 2 min, 50% and 50% for 3 min, 20% and 80% for 1 min, 10% and 90% for 2 min and then linearly changed to 34% B over 8 min, and turned back to initial condition 90% A and 10%B and washed until 15.00 min. The column temperature was adjusted at 35.0 °C. The flow rate was 0.3 ml/min and the injection volume was 5 μl. Mass spectrometry was performed in negative electrospray mode using a high-resolution mass spectrometer synapt G2 S HDMS (Waters, Manchester, UK) with a TOF-detector with linear dynamic range of at least 5000:1. The desolvation gas (45 °C, 647.0 L/h) and the nebulizer gas (6.0 bar) were nitrogen. The cone gas had a flow of 52 L/h. The capillary voltage was 2.52 kV and the source temperature 90 °C. The analyzer mode was set at ‘resolution’ and the dynamic range at ‘extended’. The mass spectra were acquired over the range of 100–1000 Da with a spectral acquisition rate of 0.1 s per spectrum. We used 1,2-dipalmitoyl-sn-glycero-3-phosphate sodium salt (DPPA), Enzo Life Sciences, USA as a standard phosphatidic acid.

### Growth inhibition assay

To assess the effect of PA imbalance on growth rate of parasite, synchronized parasite culture at troph stage was treated with increasing concentration of propranolol (5–500 μM) and allowed to grow over one cycle, i.e., 48 h. After one cycle the parasite is freeze thawed and processed for SYBR green (Invitrogen, USA) staining. Briefly, equal volume of lysis buffer containing Tris (20 mM; pH 7.5), EDTA (5 mM), saponin (0.008%; wt/vol), and Triton X-100 (0.08%; vol/vol) is added to parasite culture. 1X SYBR green dye (Invitrogen, USA) is added to the parasite and incubated for 3 h at 37 °C. The plate is read by fluorimetry at excitation and emission wavelength of 485 and 530 nm respectively. Percent growth inhibition is calculated using the following formula:

% Growth inhibition = {(Control-treated)/(control)* 100}.

### Egress assay

To assess the effect of propranolol on parasite egress, segmented schizonts (~44–48 h post invasion (hpi)) were diluted to a final hematocrit of 2% and parasitemia of ~5% and treated with 125 and 250 μM propranolol (Sigma, USA). Untreated parasites were taken as control. Following treatment, schizonts were incubated for 2–4 h to allow rupture and release of merozoites. After 2 h, Giemsa stained smears were made from treated and untreated parasites. We counted the number of stuck schizonts in total 3000 erythrocytes. Percent egress was calculated as the fraction of schizonts ruptured in treatment and control during the incubation time considering initial number of schizonts to be 100 in number.

To calculate rings formed per schizont egress, number of schizonts (before and after treatment) and number of rings formed were counted. Number of rings formed per schizont egress is calculated using the below formula:

Number of rings formed per schizont egress **=** (Number of rings)/(Number of schizonts before treatment-number of schizonts after treatment)

### Secretion assay

To analyse the secretion from intraerythrocytic merozoites, schizonts (~44–46 hpi) were treated with 1 mM EGTA, 50 μM BAPTA-AM and DMSO control for 4–6 h at 37 °C. PV and RBC fractions of schizonts were collected by 0.05% saponin treatment and probed for the presence of PfPLP1,EBA-175,MSP-1, by western blot analysis using anti-PfPLP1,AMA-1,NAPL mouse sera. To analyse secretion of PfPLP1 from free merozoites, merozoites were isolated as described previously37 and incubated with iRPMI, iRPMI containing 10 µm PA, iRPMI containing 50 μM BAPTA-AM and iRPMI containing propranolol for 15 min at 37 °C. Following incubation, supernatants were separated by centrifugation and used for detection of PfPLP1 and AMA1 by western blotting using anti-PfPLP1 mouse sera (1:200), anti-AMA1 rabbit sera (1:2000) or anti-NAPL rabbit sera (1:2000) followed by horse radish peroxidase (HRP)-conjugated anti-mouse or anti-rabbit IgG goat sera (Sigma, USA).

### Live-cell video microscopy during egress

To study role of phosphatidic acid in egress, purified *P. falciparum* schizonts were loaded with propranolol, placed in coverslip bottom petriplates and observed under a confocal microscope equipped with a temperature-controlled stage (Nikon A1R). DIC and fluorescent images (505–550 bandpass filter) were captured using a ×100, 1.4 numerical aperture lens. 12-bit time-lapse images of egress events were captured.To test host cell membrane permeabilization during egress, purified *P. falciparum* schizonts were placed in coverslip bottom petriplates along with 100 μM Phalloidin Alexa 488 and observed for rupture of schizonts under confocal microscope. DIC and fluorescent images (488 bandpass filter) were captured.

### Statistical analysis

The data for all the assays are expressed as the mean ± standard deviation (SD) of three independent experiments done in triplicates. Student’s *t*-test was performed to calculate the p values, where p < 0.05 was taken as significant.

## Supplementary information

Supplementary Data

Egress of untreated schizonts

Egress of Propranolol treated schizonts

Egress of untreated schizonts

Egress of Propranolol treated schizonts
