# Supplementary material for: Phosphatidic acid homeostasis regulated by a type-2 phosphatidic acid phosphatase represents a novel druggable target in malaria intervention
Source: Cell Death Discov. 2019 Jun 24;5:107. doi: 10.1038/s41420-019-0187-1 (PMC6591222; doi:10.1038/s41420-019-0187-1)
Supplement: Supplementary file 1 — Supplementary Data [file 41420_2019_187_MOESM1_ESM.pdf]

Phosphatidic acid homeostasis regulated by a type-2 phosphatidic acid phosphatase represents a novel druggable target in malaria intervention

Raj Kumar Sah[1#], Swati Garg[2#], Poonam Dangi[1,3#], Kalaiarasan Ponnusamy[4] and Shailja Singh[1, 2*]

[1]Special Center for Molecular Medicine, Jawaharlal Nehru University, New Delhi, India.

[2]Department of Life Sciences, School of Natural Sciences, Shiv Nadar University, Gautam Buddh Nagar, India.

[3] Current Affiliation: Département de biochimie, Faculté de Médecine, Université de Montréal, Montreal, Canada.

[4]School of Biotechnology, Jawaharlal Nehru University, New Delhi, India

[#] Equal Contribution

*To whom correspondence may be addressed at: Dr. Shailja Singh, Associate Professor, Special Center for Molecular Medicine, Jawaharlal Nehru University, New Delhi, India. E-mail: shailja.jnu@gmail.com

Running title: PfPAP2 is an anti-malarial drug target

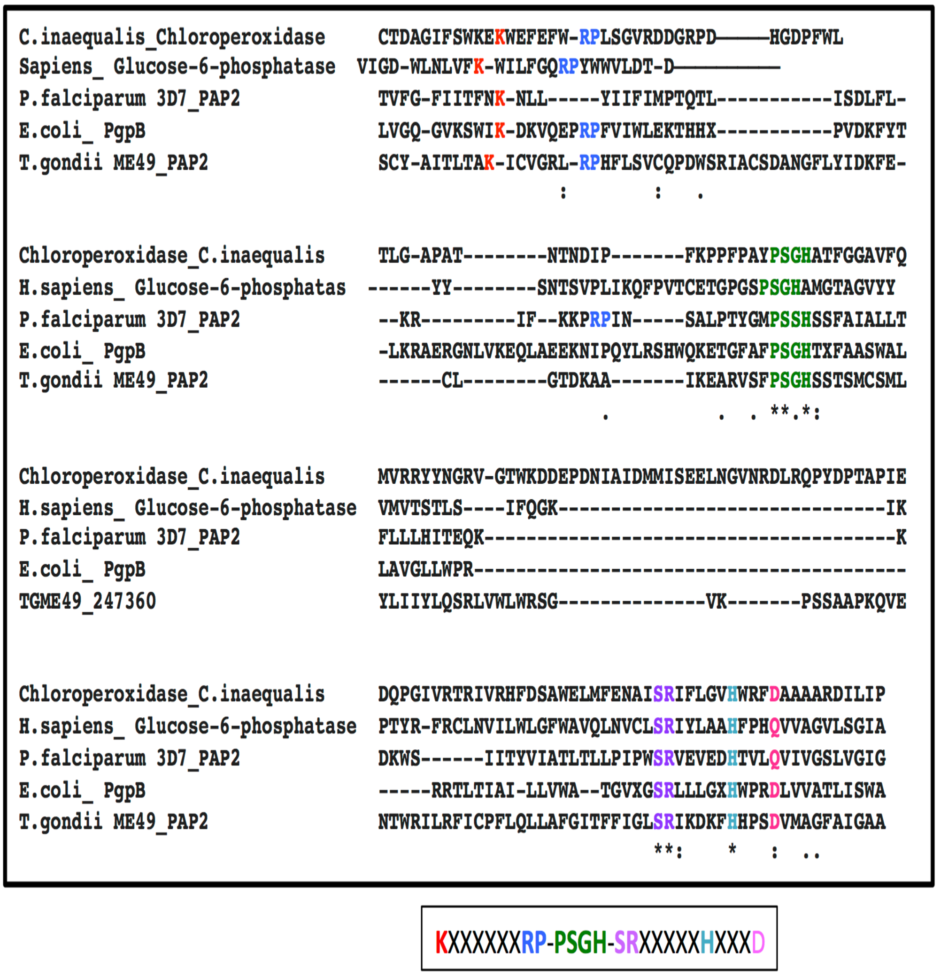

Supplementary figure 1: Multiple sequence alignment of acid phosphatase domain of PfPAP2 with acid phosphatase domain of other known members of PAP2 superfamily. KXXXXXXRP-PSGH-SRXXXXXHXXXD is the characteristic signature motif of acid phosphatase domain, which is conserved across all the members of PAP2 superfamily

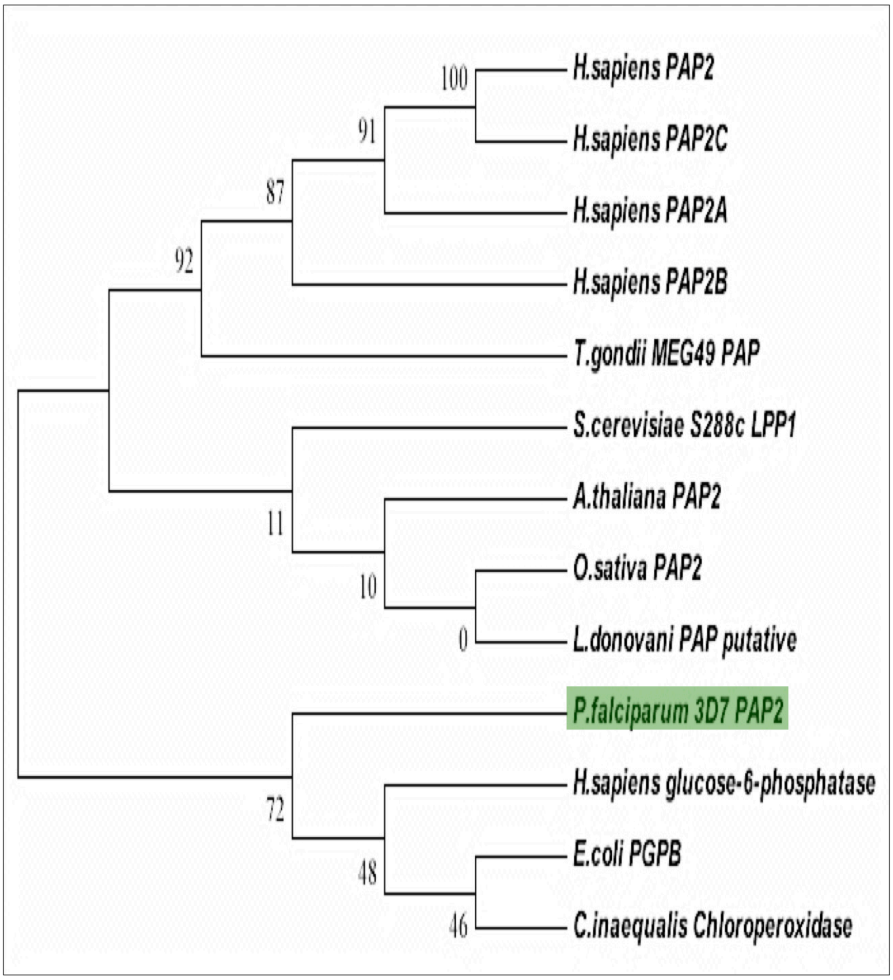

Supplementary figure 2: Phylogenetic analysis of PfPAP2. Phylogenetic analysis was performed using protein sequences of known members of PAP2 superfamily using Maximum Likelihood method. Phylogenetic tree was constructed using MEGA 7 software with bootstrap value of 500. Out of all the protein sequences analyzed PfPAP2 is most closely related to the *E.coli* PAP2, ecPgpB.

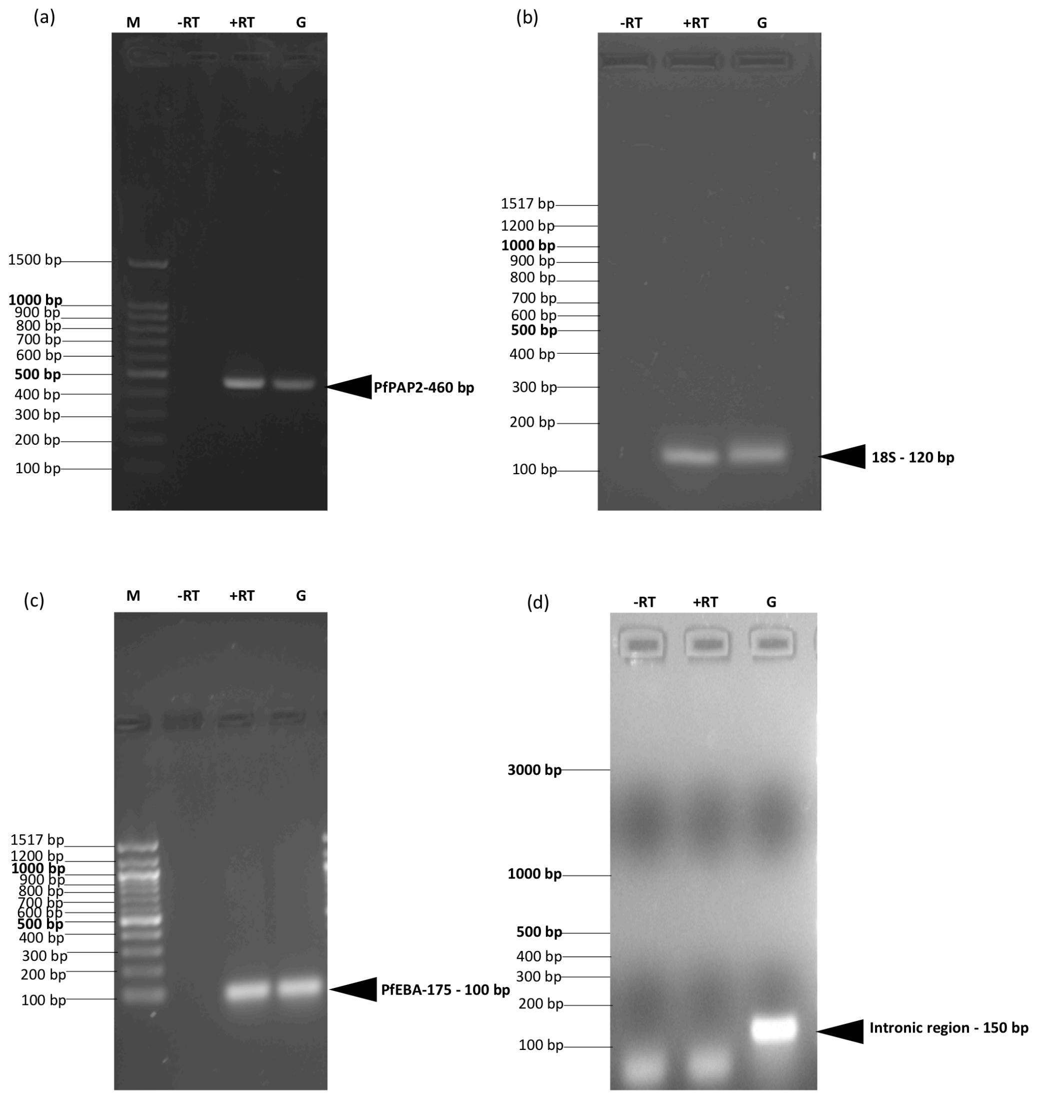

Supplementary figure 3: RT-PCR analysis of PfPAP2 and control genes. (a) A transcript of 460 bp in size confirmed expression of PfPAP2. (b). A 120 bp amplicon of 18S rRNA transcript served as an internal control. (c) PfEBA-175 was used as a late blood stage marker. An amplicon of 100 bp in size confirms EBA-175 expression. (d) Intronic primers were used to amplify a 150 bp region, served as a negative control for genomic DNA contamination.

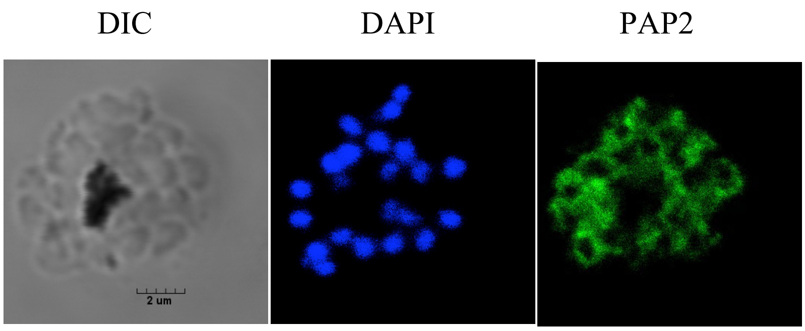

Supplementary figure 4: Immunofluorescence assay of mature schizonts using anti-PfPAP2-peptide antibody. The staining of PfPAP2 is similar to the staining revealed by anti-PfPAP2 antibody.

(a)

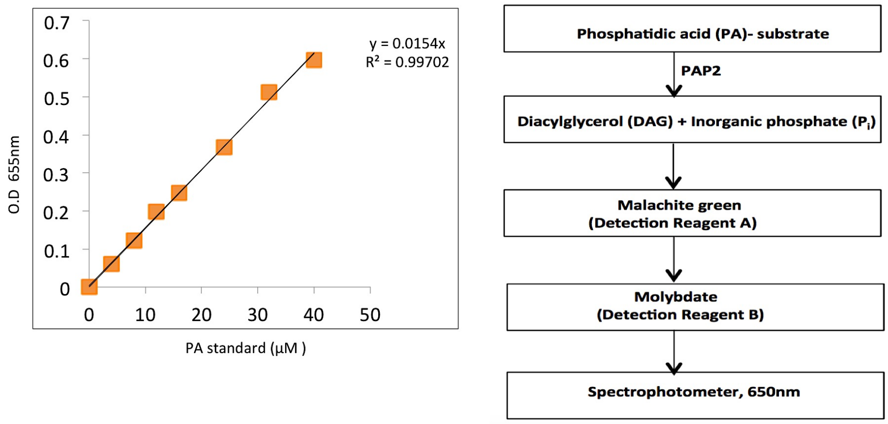

(b)

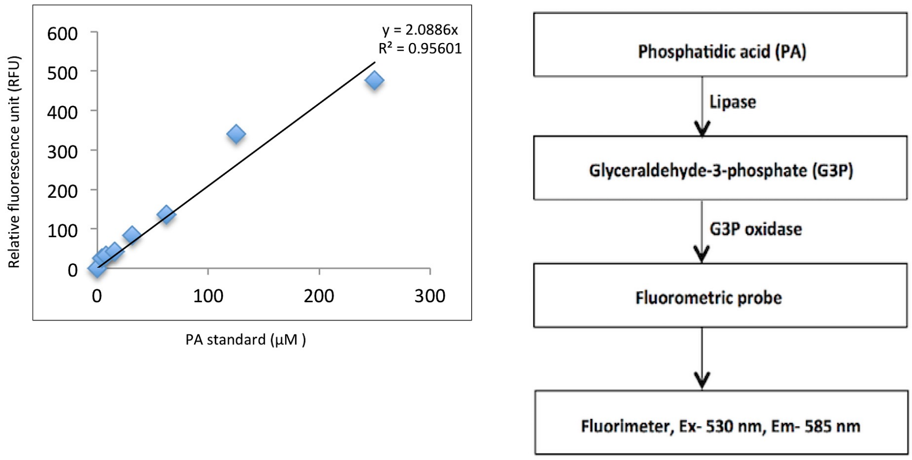

Supplementary figure 5: Standard curve and schematic representation of the principle for Malachite Green assay (Bioassays systems, USA) (a) and Total PA estimation assay kit (Cell Biolabs, Inc., USA) (b).

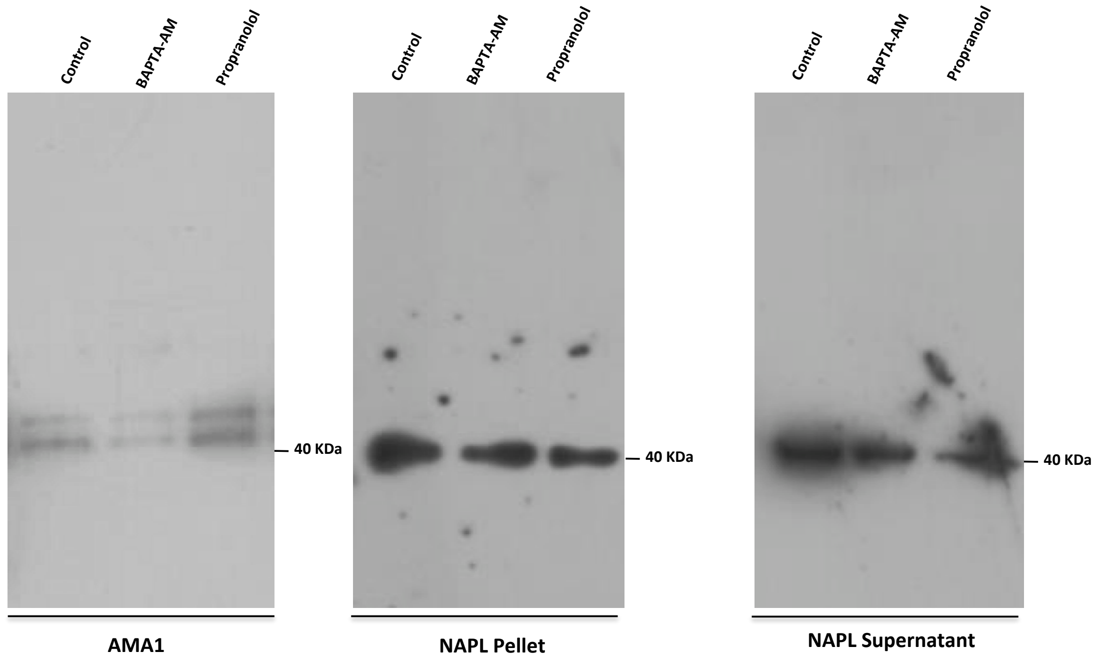

Supplementary figure 6: Full blots of merozoite secretion assay for Figure 4 (e).
